# Coronavirus lockdown and its impact on mental health of general population

**DOI:** 10.1192/j.eurpsy.2021.713

**Published:** 2021-08-13

**Authors:** M. Dhemaid, W. Abbes, C. Bagane, A. Jradi, S. Hafi, L. Ghanmi

**Affiliations:** 1 Psychiatry, regional hospital of Gabes, Gabes, Tunisia; 2 Prehospital Emergency Care Service, regional hospital of Gabes, Gabes, Tunisia

**Keywords:** General population, COVID-19, Anxiety, Depression

## Abstract

**Introduction:**

On March, Tunisian government imposed lockdown measures on cities to contain the COVID19 outbreak. Media coverage, social distancing, quarantine and isolation led to a global atmosphere of anxiety and depression.

**Objectives:**

To assess the level of anxiety and depression among citizens of southern Tunisia and theirs associated factors.

**Methods:**

We conducted a cross-sectional, descriptive and analytical online-based survey, from April 19, 2020, to May 5, 2020 on 331 citizens living in south of Tunisia. During this period, the total confirmed cases of COVID-19 exceeded 900 in Tunisia. We used a self-administered anonymous questionnaire containing citizen’s sociodemographic and clinical data. Hospital Anxiety and Depression Scale (HAD) validated in the Tunisian dialectal version was used to assess anxiety and depression. Data were analysed using SPSS version 21.

**Results:**

The 331 Participants were males (35%), singles (43,2%),aged between 20 and 40 years old(71%). From them, 37.5 % were suffering from anxiety and 42% of them from depression. Anxiety was correlated to the personal history of anxiety (p<10^-3^), the depression (p<10^-3^), the fear of contamination (p<10^-3^), the increased consumption of coffee and tea (p=0.005) and sleep disorders (p<10^-3^). Meanwhile, depression was associated to a past psychiatric history (p=0.001), a personal experience of psychological violence (p=0.011), increased cannabis use (p=0.011) and a broken sleep (p=0.007).

**Conclusions:**

Our study identified a high prevalence of adverse psychological symptoms experienced by Tunisian citizens during this first wave of virus spread. Mitigating coronavirus effect on mental health is becoming an international public health priority.

